# Assessment of Sublethal Effects of Neonicotinoid Insecticides on the Life‐History Traits of 2 Frog Species

**DOI:** 10.1002/etc.4511

**Published:** 2019-08-06

**Authors:** S.A. Robinson, S.D. Richardson, R.L. Dalton, F. Maisonneuve, A.J. Bartlett, S.R. de Solla, V.L. Trudeau, N. Waltho

**Affiliations:** ^1^ Environment and Climate Change Canada, Ecotoxicology and Wildlife Health Division Ottawa Ontario Canada; ^2^ Department of Biology Carleton University Ottawa Ontario Canada; ^3^ Environment and Climate Change Canada, Ecological Assessment Division Gatineau Quebec Canada; ^4^ Environment and Climate Change Canada, Aquatic Contaminants Research Division, Burlington Ontario Canada; ^5^ Environment and Climate Change Canada, Ecotoxicology and Wildlife Health Division, Burlington Ontario Canada; ^6^ Department of Biology University of Ottawa Ottawa Ontario Canada

**Keywords:** Clothianidin, Thiamethoxam, Amphibians, Pesticides, Ecotoxicology

## Abstract

Neonicotinoid insecticides are used extensively in agriculture and, as a consequence, are now detectable in nearby aquatic environments. Few studies have evaluated the effects of neonicotinoids on amphibians in these aquatic environments. In the present study, we examined the effects of 2 commercial formulations of neonicotinoids (active ingredients clothianidin and thiamethoxam) on survival and life‐history traits of wood frogs (*Lithobates sylvaticus*) and northern leopard frogs (*Lithobates pipiens*). We used artificial pond mesocosms to assess the effects of these neonicotinoids, at nominal concentrations of 2.5 and 250 µg/L, on amphibian larval development through metamorphosis. We found no differences between controls and neonicotinoid exposure for any of the endpoints assessed for either wood frogs or leopard frogs. The present study suggests that concentrations meeting or exceeding observed levels of clothianidin and thiamethoxam in surface waters will not directly affect metamorphosis in 2 amphibians. *Environ Toxicol Chem* 2019;38:1967–1977. © 2019 Her Majesty the Queen in Right of Canada. *Environmental Toxicology and Chemistry* published by Wiley Periodicals LLC on behalf of SETAC.

## INTRODUCTION

Globally, neonicotinoid insecticides are one of the most widely used classes of insecticides (Jeschke et al. 2011; Goulson 2013; Douglas and Tooker 2015). Imidacloprid, clothianidin, and thiamethoxam are 3 of the most commonly used of the 7 readily available neonicotinoids (Simon‐Delso et al. 2015). These systemic insecticides resemble the structure of nicotine and therefore act on insect nervous systems by targeting nicotinic acetylcholine receptors (Anderson et al. 2015; Simon‐Delso et al. 2015). Neonicotinoids degrade in shallow waters when exposed to ultraviolet light (Bonmatin et al. 2015; Morrissey et al. 2015); therefore, fast degradation under laboratory conditions is predicted. However, neonicotinoids have been found to persist in aquatic environments up to 1 yr postapplication where penetration of light is reduced because of plant growth, water turbidity, depth, and terrestrial shading (Gibbons et al. 2014; Main et al. 2014, 2016; Bonmatin et al. 2015; Morrissey et al. 2015; Main et al. 2016). Furthermore, depending on how neonicotinoids are applied, only a small percentage (2–28%) of the applied neonicotinoid is absorbed by target plants (e.g., imidacloprid; Sur and Stork 2003), suggesting that 78 to 98% of the application may enter into the surrounding soils and nearby water bodies (Anderson et al. 2015; Miles et al. 2017). Consequently, nontarget species living in these receiving aquatic habitats may unintentionally be exposed to deleterious concentrations of neonicotinoids through runoff following rainfall or irrigation events, overspray, greenhouse wastewater drainage, snowmelt, or decomposition of treated seeds (Main et al. 2014, 2016; Bonmatin et al. 2015; Morrissey et al. 2015). Morrissey et al. (2015) reported that in the Canadian prairies and Texas high plains, maximum neonicotinoid concentrations in wetlands were 3.1 and 225 µg/L for clothianidin and thiamethoxam, respectively. Recently, Metcalfe et al. (2019) detected imidacloprid, thiamethoxam, and clothianidin at maximum concentrations of 0.74 to 1.61 µg/L in 18 agricultural watersheds that drain into the lower Great Lakes in Canada. These concentrations exceed the recommended thresholds for protecting sensitive aquatic invertebrate species of 0.2 and 0.025 µg/L for short‐term peak and long‐term neonicotinoid exposures, respectively (Morrissey et al. 2015). These concentrations also exceed currently established guidelines for imidacloprid in various countries (e.g., 0.008 µg/L The Netherlands, 0.230 µg/L Canada, 0.067 µg/L Europe; additional information can be found in Morrissey et al. 2015). Because of overlapping toxicity and the same mode of action, it was previously expected that imidacloprid, clothianidin, and thiamethoxam had similar toxicity thresholds (Morrissey et al. 2015); however, it has been shown more recently that acute toxicity can vary between neonicotinoids (Raby et al. 2018).

Neonicotinoids have a range of sublethal effects on nontarget terrestrial and aquatic wildlife (Gibbons et al. 2014). White‐crowned sparrows (*Zonotrichia leucophrys*) fed 4 imidacloprid‐treated seeds per day over 3 d experienced significant impairment in their ability to orient themselves, in addition to declines in fat stores and body mass, impaired migration, and decreased condition that could lead to overall fitness loss (Eng et al. 2018). Furthermore, several species of fish exposed to 30 to 1340 µg/L imidacloprid and 20 000 µg/L clothianidin experienced physiological stress and DNA damage (Gibbons et al. 2014). Various studies have also evaluated direct effects of imidacloprid exposure to amphibians. Wood frogs (*Lithobates sylvaticus*) had reduced mortality and a slight delay in metamorphosis under chronic exposure to 10 and 100 µg/L of imidacloprid (Robinson et al. 2017). A reduction in survival for northern cricket frogs (*Acris crepitans*) was found with exposure to extreme concentrations of imidacloprid (i.e., 9000 µg/L; Ade et al. 2010). In addition, Blanchard's cricket frogs (*Acris blanchardi*) chronically exposed to 1000 µg/L of imidacloprid had larger mass at metamorphosis compared to controls (Boone 2018). However, no adverse effects of neonicotinoid exposure were detected in green frogs (*Lithobates clamitans*) exposed to imidacloprid up to 9000 µg/L (Ade et al. 2010). Hence, there is potential for species‐specific responses and sensitivities to neonicotinoids. These research efforts in combination with the widespread use and persistence of neonicotinoids have raised both academic and public concern regarding the effects of chronic exposure to nontarget species, including vertebrates.

Amphibians are of particular concern because according to the 2001 analysis of amphibians on the International Union for Conservation of Nature and Natural Resources’ Red List, 32% of global amphibian species are indicated as threatened or endangered and 42% of all species are in decline (Stuart et al. 2004). Chronic exposure to pesticides in aquatic environments receiving agricultural runoff has been suggested as a contributing factor to these declining amphibian populations (Davidson 2004; Sparling et al. 2015; Grant et al. 2016). That surface water concentrations of neonicotinoids peak in the spring (Struger et al. 2017) during the period of breeding for numerous amphibian species indicates that exposure is highest during oviposition and subsequent embryonic development. During metamorphosis, amphibians undergo a complete reorganization of internal structures and tissues, requiring properly functioning endocrine pathways (Sillar et al. 2008). These endocrine pathways can readily be disrupted by environmental contaminants including pesticides, resulting in intersex or other developmental complications (Vatnick et al. 2004; Navarro‐Martín et al. 2014).

Chronic exposure to environmental contaminants affecting amphibian life‐history traits could have fitness‐related or genetic consequences for individuals and ultimately for populations (Semlitsch et al. 2000; Fasola et al. 2015). Contaminants may change the life‐history strategies of amphibians by altering the trade‐offs between life‐history traits, some of which may be attributable to energetic costs in response to toxicants (Fasola et al. 2015). For example, glyphosate at concentrations of 2.9 mg acid equivalents/L significantly increased time to metamorphosis in wood frogs, potentially extending tadpole exposure to aquatic predators or stranding tadpoles in ephemeral pools (Navarro‐Martín et al. 2014). Thus, survival and life‐history traits are important indicators of environmental contamination, reinforcing the view that amphibians are valued as bioindicators of ecosystem health as a whole (Hopkins 2007).

To evaluate effects of neonicotinoids on the life history of amphibians, we selected 2 native Canadian frog species, the wood frog (*L. sylvaticus*) and the northern leopard frog (*L. pipiens*, formerly *Rana pipiens*). Wood frogs and northern leopard frogs are found in agriculture‐associated aquatic environments, and thus, these species are likely exposed to neonicotinoids (Knutson et al. 2004; Boczulak et al. 2017). Specifically, we assessed the impact of neonicotinoid exposure on frog life‐history traits with the aid of outdoor mesocosms in a controlled experiment, using concentrations of clothianidin and thiamethoxam at both 2.5 and 250 µg/L. These test concentrations meet or exceed the maximum concentrations of clothianidin and thiamethoxam observed in surface waters (3.1 and 225 µg/L, respectively; Morrissey et al. 2015). Although vertebrate toxicity data are limited and generally not known for these 2 neonicotinoids, our test concentrations were significantly less than the concentrations of imidacloprid estimated to be lethal to amphibians (i.e., 52 600–366 000 µg/L; Feng et al. 2004; Sánchez‐Bayo 2012; Gibbons et al. 2014; Pérez‐Iglesias et al. 2014; Ruiz De Arcaute et al. 2014).Thus, we targeted sublethal concentrations for the chronic exposure of clothianidin and thiamethoxam; and although frog survival was assessed, the focus of the present study was investigating sublethal effects on metamorphosis and sex differentiation.

## METHODS

### Experimental setup

A 50‐mesocosm array of experimental artificial ponds (Rubbermaid®; 132 × 78 × 63 cm) was established in a fenced‐off research garden at Carleton University in April to May 2015. The 50 experimental mesocosms were divided between the 2 frog species in two 5×5 arrays (i.e., one control plus 4 treatments with 5 replicates [blocks] each), for a total of 25 mesocosms per species. The experiments were run simultaneously for both species, with a delay of 14 d between the wood frog and leopard frog exposures, because of the seasonal breeding constraints for both species. These mesocosms were filled with 300 L of tap water and 333.3 ± 5.0 g of mixed pesticide‐free deciduous leaf litter (following Robinson et al. 2017). Stock mesocosms for *Daphnia magna*, *Ceriodaphnia* sp., and both wood frog and leopard frog tadpoles (individually for each species) were established and maintained under similar conditions as the experimental mesocosms (but without the addition of leaf litter and aeration). Stock mesocosms enabled natural algae to establish and water quality to be monitored from 29 April to 27 May (for each stock mesocosm during this time period the average temperature range was 16.0 ± 1.9 °C to 17.2 ± 3.5 °C, the average dissolved oxygen range was 0.6 ± 0.2 mg/L [*Daphnia* spp. tanks] to 8.0 ± 2.2 mg/L [tadpole spp. tanks], and the average pH range was 7.3 ± 0.2 to 7.5 ± 0.4).

Prior to the addition of tadpoles, experimental mesocosms were inoculated with 1 L of stock water containing *D. magna*, 1 L of stock water containing *Ceriodaphnia* sp., and 1 L of water from a wetland not associated with agriculture (simulating a pond environment containing zooplankton, microbes, and algae). Nitex mesh cages (35×60 cm, 500 μm mesh; Harris et al. 2001) were also placed in experimental mesocosms, and all experimental mesocosms were aerated using air stones to maintain dissolved oxygen throughout the experiment at>3.5 mg/L (>40%; Organisation for Economic Co‐operation and Development 2009) following temporarily low <2 mg/L (<20%) dissolved oxygen levels in the mesocosms prior to the addition of tadpoles (likely attributable to seasonally high temperatures and decomposition of leaf litter; Supplemental Data, Table S1). All experimental and stock mesocosms were covered with 40% shade cloth to prevent unwanted organisms from entering or frogs from escaping (Supplemental Data, Figure S1). The experimental mesocosms were left to acclimatize for up to 7 d prior to adding the tadpoles.

Male and female adult wood and leopard frogs were collected from remote wetlands not associated with agricultural fields near Bishops Mills, Ontario, Canada (44.87366, –75.70455) from late March to mid‐April. The adult wood frogs were transported to the University of Ottawa and allowed to spawn naturally under laboratory conditions, as described in Robinson et al. (2017). Six wood frog egg masses were moved outdoors to 2 stock mesocosms within the mesocosm complex on 30 April, mixed for genetic diversity with 3 egg masses per mesocosm, and then left to hatch and develop into free‐swimming tadpoles at Gosner stage 25 (Gosner 1960; hatching occurred between 4 and 6 May). Northern leopard frog eggs were collected from an independent breeding experiment similarly conducted in outdoor mesocosms, where adult leopard frogs were injected with a hormone mixture comprised of a gonadotropin‐releasing hormone agonist and a dopamine antagonist and allowed to spawn (Vu et al. 2017; clutches were laid between 6 and 9 May). A subsample of the fertilized leopard frog egg masses was used for our experiment, where 16 partial egg masses were placed in stock mesocosms at a density of 4 partial egg masses per stock mesocosm and similarly left to hatch and develop to Gosner stage 25; hatching began on 12 May). Tadpoles were held in stock mesocosms at low to medium density for 7 to 9 d (wood frogs and leopard frogs, respectively) before being transferred to their respective experimental mesocosm.

On 11 May (for wood frogs) and 21 May (for leopard frogs) experimental tadpoles (*n* = 20) were added to the Nitex mesh cages within experimental mesocosms and held together for up to 7 d under the same ambient conditions as the stock tanks to acclimatize to the mesocosms before the addition of neonicotinoid treatments (first dose administered 14 May for wood frogs and 28 May for leopard frogs). Tadpoles were photographed with a scale bar for later body measurements using ImageJ image analysis software (Ver 1.48; US National Institutes of Health). The purpose of the Nitex mesh cages was to hold tadpoles to collect corticosterone concentrations (data not included in the present study; Gavel et al. 2019) before they were released into the full 300‐L mesocosm. The tadpoles were exposed to one dose of neonicotinoid treatment (see section *Neonicotinoid insecticides—Mesocosm treatments*) and fed on algae while being held within the Nitex mesh cages. After 6 d of exposure to the neonicotinoids, we collected corticosterone for a separate study (Gavel et al. 2019) and recorded the number of tadpoles that were alive. Percent survival was ≥ 69% for wood frogs and ≥ 96% for leopard frogs. All surviving tadpoles (without replacement) were subsequently released (20 May for wood frogs and 2 June for leopard frogs) into the full 300‐L experimental mesocosms for the duration of the experiments. When the tadpoles developed hind limbs, we provided semiterrestrial surfaces to be used as “rafts” (1‐cm‐thick slices of PoolNoodle® and 20 × 20 cm high‐density polyethylene; see Dalton et al. 2014). Tadpoles were monitored daily for forelimb development and complete tail reabsorption and immediately removed and processed (see section *Amphibian assessment—Sublethal effects*) when they reached Gosner stage 46. Number of days to metamorphosis was calculated from the day tadpoles were added to the mesocosms (i.e., 11 and 21 May for wood frogs and leopard frogs, respectively) and the day they were removed from the mesocosms when they reached Gosner stage 46.

To monitor mesocosm water quality, we recorded weekly measures of temperature (°C), pH, and dissolved oxygen (mg/L; Supplemental Data, Table S1). Dissolved oxygen and temperature were measured using a YSI Environmental ProODO Handheld Optical Dissolved Oxygen Meter, and pH was measured using a compact pH meter (LAQUAtwin B‐71x; Horiba Scientific).

### Neonicotinoid insecticides—Mesocosm treatments

We assessed the sublethal effects of 2 commercial formulations of neonicotinoid insecticides on wood and leopard frogs: Bayer's clothianidin‐based Titan (600 g/L of clothianidin; Bayer CropScience; Chemical Abstracts Service [CAS] no. 210880‐92‐5), and Syngenta's thiamethoxam‐based Actara® (240 g/L of thiamethoxam; CAS no. 153719‐23‐4). Thiamethoxam stock solutions were prepared by diluting 0.1 and 10 mL of the commercial formulation into 800 mL of reverse osmosis water. Clothianidin stock solutions were prepared by diluting 0.04 and 4 mL of the commercial formulation in 800 mL of reverse osmosis water. Nominal stock solution concentrations were 0.03 and 3 g/L for each active ingredient and were stored in amber glass bottles in the dark at 4 °C throughout the experimental period.

We used a random block design to assign treatments to the experimental mesocosms (300 L), with one treatment represented in each of the 5 blocks (blocks ran north–south to account for possible shading effects of neighboring mesocosms; Supplemental Data, Figure S2). We incorporated blind exposures into our design to ensure nonbias in data collection by randomly assigning a color code to represent one of the 5 treatments: 1, control (reverse osmosis water); 2, low clothianidin (2.5 µg/L); 3, low thiamethoxam (2.5 µg/L); 4, high clothianidin (250 µg/L); and 5, high thiamethoxam (250 µg/L). Although neonicotinoids have been noted to persist in the environment for long periods of time, our mesocosms were dosed weekly for the first 2 wk of exposure, followed by applications every 2 wk. The neonicotinoids were applied to the mesocosms to maintain chronic exposures throughout the study period and to replicate pulse input events such as rainfall and spring runoff (Struger et al. 2017). Wood frog mesocosms received 10‐mL aliquots of designated treatment stock solutions on 14 May, followed by 25‐mL aliquots on 21 May and 4 and 18 June. The volume used to dose the mesocosms was increased from 10 to 25 mL using the same stock solutions, to compensate for a perceived low percent recovery of neonicotinoids that was identified from our first set of stock solutions; thus, our nominal concentrations of 2.5 and 250 µg/L reflect the increase in dose solutions following the low percent recovery (see details in *Neonicotinoid analyses* in Supplemental Data and Tables S2 and S3). Leopard frog mesocosms received 25‐mL aliquots of designated treatment stock solutions on 28 May, 4 and 18 June, and 2 July. In all cases, dosing aliquots were poured over the surface of the mesocosm in an “S” pattern, followed by rinsing the dispensing centrifuge tube with mesocosm water and following the same “S” pattern to promote mixing of the solution. The aeration in each mesocosm also helped to ensure that the treatment solution was well distributed throughout the mesocosm.

Water samples were collected from the mesocosms several times during the exposure to confirm clothianidin and thiamethoxam concentrations. Specifically, water samples were first collected from all 50 mesocosms 1 h after the first dosing to confirm concentrations in the mesocosms at the beginning of the exposure period. Weekly water samples were then collected for the duration of the experiment from 5 randomly selected mesocosms that consisted of one replicate from each treatment for both wood frog and leopard frog experiments. Water samples were collected from the center of the mesocosms at mid–water column in 500‐mL amber glass bottles and stored at 4 °C in the dark to prevent photodegradation. Chemical analyses were conducted by Laboratory Services, National Wildlife Research Centre (Environment and Climate Change Canada) following a previously published method (Robinson et al. 2017). The chemical analyses were performed on a high‐performance liquid chromatograph (1200 Series; Agilent Technologies) with a tandem mass spectrometer (API 5000 Triple Quadrupole Mass Spectrometer and Turbo V™ Ion Source; AB Sciex) following the same methods as Robinson et al. (2017) except that solid‐phase extraction was not used.

The quality assurance system for the analytical method included establishing a calibration curve with 8 levels and an *R* ^2^ > 0.995 for each set of samples. Analytical precision was assessed using sample duplicates, where the difference between samples was <5%. Analytical accuracy was assessed using commercially prepared second source standards, where the recoveries between experimental and expected values were between 85 and 115%. The limits of detection for clothianidin and thiamethoxam were 0.05 and 0.03 µg/L, respectively; and the limits of quantification for clothianidin and thiamethoxam were 0.17 and 0.09 µg/L, respectively.

### Amphibian assessment—Sublethal effects

Frogs were removed from the mesocosms when they reached Gosner stage 46 (i.e., at completed metamorphosis; Gosner 1960). Removed wood and leopard frogs were anesthetized by immersion in 0.01% buffered solution of tricaine methane sulfonate (MS‐222; Sigma‐Aldrich), blotted dry, weighed ( ± 0.01 g; A&D, Newton series EJ‐1500), and photographed (Sony DSC‐RX100) with a scale bar to determine snout‐to‐vent length (mm) using ImageJ. Intraobserver variability in snout‐to‐vent length measurements was assessed by measuring 3 randomly selected metamorphs and tadpoles 13 to 20 different times without reference to previous measurements. An acceptable level of precision was found (Hayek et al. 2001), where the mean coefficient of variation for snout‐to‐vent length was 0.05% for wood frogs and 0.02% for leopard frogs (to account for different observers).

Gosner stage 46 leopard frogs were euthanized by immersion in 1% buffered solution of MS‐222 (Sigma Aldrich) and dissected, and the gonads were identified as either male or female by visual inspection immediately following prior anesthesia (Supplemental Data, Figure S3; Witschi 1931). The leopard frog experiment was terminated prior to all animals completing metamorphosis because of time constraints. All remaining leopard frog tadpoles from the mesocosms (*n* = 105 of the 431; 24%) that did not complete metamorphosis were collected and included in the survival analyses. Developmental stage of these tadpoles according to Gosner was recorded for the individuals that survived to the end of the experimental period (28 July, experimental day 69) but had not reached Gosner stage 46. We did not dissect or determine the sex of leopard frogs that had not reached Gosner stage 46, and they were not included in calculating time to metamorphosis. For wood frogs, immediately following anesthesia, all metamorphs (last wood frog was removed from the mesocosms on 8 July) were individually housed and kept for approximately 3 wk postmetamorphosis in an environmental chamber for a separate study (Gavel et al. 2019). Following termination, all remaining wood frogs were subsequently euthanized and dissected, and the gonads were identified. All sampling and handling of animals followed the Canadian Council for Animal Care guidelines and approved protocols by the University of Ottawa Animal Care Committee (BL‐2206) and Environment and Climate Change Canada's Wildlife East Animal Care Committee (1528SR01).

### Statistical analyses

We began our analyses by confirming that there were no environmental/water quality differences in our block design among treatments. To do so, we used nonparametric Kruskal‐Wallis tests to examine differences in temperature, dissolved oxygen, and pH among treatments for early, mid, and late time periods during each experiment (Supplemental Data, Table S1). We used independent Kruskal‐Wallis comparisons because the data did not meet the assumptions of parametric tests.

Next, we determined if treatment (i.e., neonicotinoid type and concentration) affected the 1) survival and 2) life‐history traits of our 2 amphibian species. We modeled treatment effects on survival and life‐history traits using general(ized) linear mixed models (GLMM) in R statistical software, Ver 3.4.0 (R Core Development Team 2017), following procedures detailed in Robinson et al. (2017). To account for the nonindependence of tadpoles within mesocosms and following our block design, mesocosms (i.e., tanks) nested within block were included as random effects in each model. Specifically, to evaluate the effects of treatment on survival, we used a GLMM with a binomial distribution and logit link function (R package lme4).

We then determined whether neonicotinoid treatment influenced the following life‐history traits: proportion of females to males, snout‐to‐vent length, mass, days to metamorphosis, and, for leopard frogs only, Gosner stage of development. To evaluate the effects on the proportion of females to males, we used a GLMM with a binomial distribution and logit link function (R package lme4). For the remaining life‐history traits, we used a GLMM with gaussian distributions and log link function (to prevent negative terms in the model; R package lme4 and lmerTest) and included survival as a fixed effect to account for density effects (Johnson et al. 2017). For the dependent variables that were count data (i.e., days to metamorphosis, Gosner stage of development), we assessed if there was overdispersion (dispersion value >1; Zuur et al. 2013) and refit the model with a different distribution if overdispersion was detected. For both days to metamorphosis and Gosner stage of development models (for leopard frogs only), a Poisson distribution using the glmmPQL package in R corrected the overdispersion in the original models. We followed Zuur et al. (2009, 2010) to explore our data for outliers and violations of homogeneity using Cleveland dot plots and box plots and multipanel scatterplots to model relationships between response variables and each continuous variable. Following data exploration, we did not remove outliers and proceeded with the analyses with the complete data set. We validated our models following Zuur et al. (2013) using residual plots, quantile–quantile plots, and density plots to assess homogeneity of variance and goodness of fit. To confirm that we had sufficient statistical power for the present study (Hayes 1987), we used Hedge's (1982) g to calculate effect sizes for mean differences in snout‐to‐vent length and days to metamorphosis that resulted in increased measures of fitness for several amphibian species. We then determined the minimum detectable effect size following Cohen (1988) to determine if we could reliably conclude that there were no biologically relevant differences among treatments. For simplicity, we used the power table for 2‐tailed *t* tests (see Table 2.3.5 in Cohen 1988).

## RESULTS

### Water quality and neonicotinoid concentrations

Our mesocosm environmental/water quality parameters were within the recommended acceptable limits for amphibians (Organisation for Economic Co‐operation and Development 2009). For the control and neonicotinoid treatments, no differences were detected in the early exposure period for any of the abiotic conditions for both the wood frog and leopard frog experiments. However, we detected slight significant differences in mean temperature in the mid (24 June) and mean pH in both the mid and late (30 June) exposure periods in the wood frog experiment (Kruskal‐Wallis χ^2^ ranged from 14.39 to 21.57, *df* = 4, *p < *0.04; Supplemental Data, Table S1). For the leopard frog experiment, no differences were detected between the controls and neonicotinoid treatments in the early or late exposure periods for any of the abiotic conditions; however, there was a slight significant difference in the mid (24 June) exposure period for pH (Kruskal‐Wallis χ^2^ = 15.1, *df* = 4 *p < *0.02; Supplemental Data, Table S1). Similar to Ade et al. (2010), these differences could be attributable to changes in photosynthesis and algal abundance as the mesocosms developed over the experimental period; however, chlorophyll or other algal metrics would need to be measured to confirm this suggestion.

We found that our measured concentrations for clothianidin and thiamethoxam in our wood frog and leopard frog mesocosms varied throughout the exposure periods (Supplemental Data, Tables S2 and S3). Although all quality assurance and quality control measures were accurate and calibration curves were confirmed, we found that the percent recoveries (compared to nominal concentrations) for a subset of our samples were low (Supplemental Data, Table S2 and S3). As a consequence, we identified a concern with a particular batch of filters (Acrodisc syringe filter [0.45 µm polyvinylidene fluoride membrane]) used during some of the earlier chemical analyses. On review of samples from the present study, as well as samples from related studies, we are confident that the samples with high recoveries are more reflective of actual measured concentrations. Further, given that the measured values, for all samples, excluding the subset with faulty filters, were close to the nominal exposure concentrations (88–128%, mean 99.6% of the 2.5 and 250 µg/L nominal concentrations; initial targeted nominal concentrations were 1 and 100 µg/L [corresponds to 14 May concentrations only, Supplemental Data, Table S2]), our nominal concentrations are more reflective of our actual exposure concentrations (see *Neonicotinoid analyses* in Supplemental Data for full details of our investigation). Irrespective, concentrations of both neonicotinoids tended to increase over the experimental period for both wood frogs and leopard frogs despite the concern with the filters (Supplemental Data, Table S3). For example, mean concentrations (± standard deviation) were 3.5 ± 0.3 and 325.7 ± 14.5 µg/L for clothianidin treatments and 3.4 ± 0.1 and 285.5 ± 20.5 µg/L for thiamethoxam treatments (4 wk after dosing; Supplemental Data, Table S2), which are 1.1 to 1.4 times the nominal concentrations. Thiamethoxam was detected at 0.04 µg/L (just above the limit of detection of 0.03 µg/L) in one of the leopard frog control mesocosms at the beginning of the experiment but was not detected in any of the following control samples. Otherwise, there was no contamination detected in any of the wood frog or leopard frog control mesocosms throughout the experiment (Supplemental Data, Table S3). For simplicity, we provide comparison and interpretation of our effect data using nominal concentrations of 2.5 and 250 µg/L for clothianidin and thiamethoxam, respectively.

### Amphibian response—Sublethal effects

Neonicotinoid exposure did not affect survival to metamorphosis or total survival for both wood frogs and leopard frogs between the controls and neonicotinoid treatments (GLMM, *z* values ranged from –0.39 to 1.29, *p* values ranged from 0.20 to 0.70; Table 1; Supplemental Data, Table S4). Specifically, for the controls, mean ( ± standard deviation) survival was 67 ± 8% for wood frogs with similar or slightly higher mean percent survival in the neonicotinoid treatments (CLO‐2.5 = 75 ± 5%, CLO‐250 = 71 ± 15%, THI‐2.5 = 74 ± 17%, THI‐250 = 70 ± 6%). For the leopard frogs, mean survival was 85 ± 7% in the controls and mean survival ranged from 83 ± 13 to 91 ± 5% for the neonicotinoid treatments (Table 1; Supplemental Data, Table S4).

**Table 1:** Results of generalized linear mixed models analyzing differences between treatments in survival of wood frogs (*Lithobates sylvaticus*) and northern leopard frogs (*Lithobates pipiens*) after chronic exposure to clothianidin or thiamethoxam in outdoor mesocosms^a^

	Wood frogs (*L. sylvaticus*)					Leopard frog (*L. pipiens*)					
	β ± SE	*z*	*p*	Variance ± SD	*n*	β ± SE	*z*	*p*	Variance ± SD	*n*	
*Survival ~ treatment + 1|Block*											
Fixed effects											
(Intercept)	0.71 ± 0.21	3.31	<0.01			1.73 ± 0.28	6.19	<0.01			
Treatment (reference: control^b^)					67/100					85/100	
CLO‐2.5	0.39 ± 0.31	1.24	0.21		75/100	–0.15 ± 0.39	–0.39	0.70		83/100	
CLO‐250	0.19 ± 0.31	0.61	0.54		71/100	0.36 ± 0.42	0.84	0.40		89/100	
THI‐2.5	0.34 ± 0.31	1.08	0.28		74/100	–0.15 ± 0.39	–0.39	0.70		83/100	
THI‐250	0.14 ± 0.30	0.46	0.65		70/100	0.58 ± 0.45	1.29	0.20		91/100	
Random effects											
(Block – intercept)				0.003 ± 0.05					0.00 ± 0.00		

^a^ Random effect of block is included in the model and denoted by “1|Block,” and the number of frogs surviving per the original sample size (*n* = 100) is indicated for each treatment.

^b^ Nominal treatment concentrations were 2.5 and 250 µg/L for clothianidin (CLO‐2.5 and CLO‐250, respectively) and thiamethoxam (THI‐2.5 and THI‐250, respectively).

β = difference and direction (positive, negative) in the treatment means compared to the mean of the control; SD = standard deviation; SE = standard error.

For both species, neonicotinoid exposure did not affect the life‐history traits between the control and neonicotinoid treatments. Specifically, the mean proportion ( ± standard deviation) of females to males, for the controls, was 32 ± 4 and 56 ± 17% (i.e., wood frogs and leopard frogs, respectively) and, for the neonicotinoid treatments, ranged from 30 ± 10 to 70 ± 18% (GLMM *z* values ranged from –0.30 to 1.57, *p* values ranged from 0.12 to 0.76; Table 2; Supplemental Data, Table S4). We also found no significant differences in snout‐to‐vent length or mass at metamorphosis between controls and neonicotinoid treatments (GLMM *t* values ranged from –32 to 0.72, *p* values ranged from 0.09 to 0.94; Table 2; Supplemental Data, Table S5). In addition, we found no significant differences between controls and neonicotinoid treatments in the time for wood frog or northern leopard frog tadpoles to metamorphose or the mean developmental stage of northern leopard frog tadpoles that did not reach metamorphosis by the end of the experimental period (GLMM *t* values ranged from –0.93 to 1.37, *p* values ranged from 0.16 to 0.93; Table 2; Supplemental Data, Table S5). Overall, when assessing survival and the conventional life‐history traits of wood frogs and leopard frogs in a mesocosm environment, we found no significant effects of clothianidin and thiamethoxam at nominal concentrations of 2.5 and 250 µg/L.

**Table 2:** Results of generalized linear mixed models analyzing differences between treatments in morphology and development (i.e., days to metamorphosis or Gosner stage) of wood frogs (*Lithobates sylvaticus*) and northern leopard frogs (*Lithobates pipiens*) after chronic exposure to clothianidin or thiamethoxam in outdoor mesocosms^a^

		Wood frogs (*L. sylvaticus*)						Leopard frog (*L. pipiens*)					
		β ± SE	*t* or *z* ^b^	*p*	*df*	VAR ± SD	*n*	β ± SE	*t* or *z* ^b^	*p*	*df*	VAR ± SD	*n*
*Proportion female ~ treatment + 1|Block*													
Fixed effects													
(Intercept)		0.78 ± 0.26	–2.97	0.003				0.10 ± 0.26	0.39	0.70			
Treatment (reference: control^c^)							21/67						34/65
CLO‐2.5		0.46 ± 0.35	1.29	0.20			31/74	0.52 ± 0.38	1.38	0.17			35/54
CLO‐250		–0.11 ± 0.37	–0.30	0.76			20/69	0.57 ± 0.37	1.57	0.12			41/62
THI‐2.5		0.42 ± 0.35	1.20	0.23			30/73	0.55 ± 0.36	1.52	0.13			42/64
THI‐250		0.22 ± 0.36	0.60	0.55			25/69	0.38 ± 0.35	1.09	0.28			45/73
Random effects (Block – Intercept)						0.00 ± 0.00						0.007 ± 0.09	
*Snout to vent length*													
*Length ~ treatment + survival + 1|Tank + 1|Block*													
Fixed effects													
(Intercept)		24.27 ± 0.90	26.83	<0.01	18.91			30.05 ± 3.86	7.78	<0.01	17.57		
Treatment (reference: control^c^)							66						64
CLO‐2.5		–0.73 ± 0.41	–1.79	0.09	15.21		74	–1.06 ± 1.03	–1.03	0.32	15.40		51
CLO‐250		–0.29 ± 0.40	–0.72	0.48	15.81		65	–0.32 ± 1.02	–0.31	0.76	14.53		61
THI‐2.5		–0.12 ± 0.41	–0.29	0.77	15.53		73	–0.09 ± 1.01	–0.09	0.93	14.44		64
THI‐250		0.12 ± 0.40	0.31	0.76	15.27		69	0.74 ± 1.03	0.72	0.48	13.96		73
Survival		–0.14 ± 0.06	–2.21	0.04	18.42			–0.20 ± 0.22	–0.89	0.38	17.10		
Random effects													
Tank (intercept)						0.30 ± 0.55						2.01 ± 1.42	
Block (intercept)						0.06 ± 0.25						0.65 ± 0.81	
Residual						1.24 ± 1.11						6.19 ± 2.49	
*Mass ~ Treatment + Survival + 1|Tank + 1|Block*													
Fixed effects													
(Intercept)		1.08 ± 0.11	9.73	<0.01	18.89			1.47 ± 0.45	3.23	<0.01	17.39		
Treatment (reference: control^c^)							66						64
CLO‐2.5		–0.08 ± 0.05	–1.58	0.13	15.18		74	–0.13 ± 0.12	–1.07	0.30	15.33		51
CLO‐250		–0.01 ± 0.05	–0.17	0.87	15.54		65	–0.07 ± 0.12	–0.57	0.58	14.35		61
THI‐2.5		0.004 ± 0.05	0.08	0.94	15.38		73	0.04 ± 0.12	0.35	0.73	14.25		64
THI‐250		–0.01 ± 0.05	–0.12	0.91	15.14		69	0.08 ± 0.12	0.63	0.54	13.64		73
Survival		–0.02 ± 0.01	–2.31	0.03	18.55			–0.02 ± 0.03	–0.70	0.49	16.80		
Random effects													
Tank (intercept)						0.005 ± 0.07						0.03 ± 0.16	
Block (intercept)						0.001 ± 0.03						0.01 ± 0.11	
Residual						0.013 ± 0.11						0.10 ± 0.32	
*Days ~ Treatment + Survival + 1|Block/Tank*													
Fixed effects													
(Intercept)		3.84 ± 0.04	106.38	<0.01	326			3.92 ± 0.07	52.52	<0.01	293		
Treatment (reference: control^c^)							67						65
CLO‐2.5		0.001 ± 0.02	0.08	0.93	15		74	0.01 ± 0.02	0.50	0.63	15		54
CLO‐250		0.02 ± 0.02	1.23	0.24	15		68	0.03 ± 0.02	1.48	0.16	15		62
THI‐2.5		–0.01 ± 0.02	–0.61	0.55	15		73	–0.01 ± 0.02	–0.60	0.56	15		64
THI‐250		0.02 ± 0.02	1.37	0.19	15		69	–0.02 ± 0.02	–0.93	0.37	15		73
Survival		0.0005 ± 0.003	0.18	0.86	15			0.01 ± 0.004	1.71	0.11	15		
Random effects													
Tank (intercept)						0.02^d^						0.02^d^	
Block (intercept)						0.005^d^						0.02^d^	
Residual						0.39^d^						0.59^d^	
*Stage ~ Treatment + Survival + 1|Block/Tank*													
Fixed effects (Intercept)								3.68 ± 0.10	36.25	<0.01	81		
Treatment (reference: control^c^)													17
CLO‐2.5								0.04 ± 0.03	1.24	0.24	14		28
CLO‐250								0.01 ± 0.03	0.17	0.87	14		25
THI‐2.5								0.02 ± 0.03	0.72	0.48	14		18
THI‐250								0.01 ± 0.04	0.29	0.77	14		17
Survival								–0.001 ± 0.01	–0.12	0.90	14		
Random effects													
Tank (intercept)												4.41e‐07^d^	
Block (intercept)												6.19e‐08^d^	
Residual												0.60^d^	

^a^ Random effects of block and tank included in the models are denoted by “1|Block” and “1|Tank,” respectively; survival was included as a fixed effect to account for density effects, and sample size for each treatment is indicated (*n*) as well as the number of females per the number that were sexed.

^b^ Wald *z* for proportion female and *t* statistic for the remaining life‐history trait models.

^c^ Nominal treatment concentrations were 2.5 and 250 µg/L for clothianidin (CLO‐2.5 and CLO‐250, respectively) and thiamethoxam (THI‐2.5 and THI‐250, respectively).

^d^ Random effects standard deviation value, using the glmmPQL package in R.

β = difference and direction (positive, negative) in the treatment means compared to the mean of the control; *df* = degrees of freedom; SD = standard deviation; SE = standard error; VAR = variance.

Hedge's g ranged from 0.7 to 3.4, where we used mean, standard deviation, and sample size values reported by Smith (1987), Berven and Gill (1983), and Semlitsch et al. (1988). Hence, we considered 0.7 to be the minimum biologically relevant effect size for the present study. Following the power table for 2‐tailed *t* tests (see Table 2.3.5 in Cohen 1988) and using our smallest treatment sample size of 65, α = 0.05, and a power of 98% (or β = 0.02), we could reliably detect an effect size of 0.7 or greater.

## DISCUSSION

Contaminants can pose a significant risk to amphibian populations through aquatic exposure from multiple sources, and there is some evidence that pesticides used in modern agricultural practices are contributing to the global decline of amphibians (Davidson 2004; Sparling et al. 2015; Grant et al. 2016). Amphibians are frequently used as biological indicators of ecosystem health by investigating sublethal effects of various contaminants on life‐history traits (Brodeur et al. 2013; Wood and Welch 2015). The present study focused on assessing some conventional life‐history traits that influence fitness as a preliminary assessment for potential direct toxic effects of neonicotinoids on amphibians that are likely exposed in surface waters close to agricultural activity. Days to metamorphosis and body size are sensitive to environmental conditions that can have fitness‐related consequences (Chelgren et al. 2006). For example, early metamorphosis can affect frogs emigrating to terrestrial environments that may not yet have favorable conditions for survival, whereas delayed metamorphosis can strand tadpoles in temporary pools, increasing their mortality and vulnerability to predation (Chelgren et al. 2006; Navarro‐Martín et al. 2014). Body size and locomotion capability are also correlated and can result in smaller frogs being less able to evade predators effectively because they can only jump short distances during a predator attack (Calsbeek and Kuchta 2011; Kowalski et al. 2018). Furthermore, large shifts in the sex ratio of frogs could reduce reproductive success (Lambert 2015; Lambert et al. 2017). Thus, conventional life‐history traits that have fitness‐related consequences are useful as an initial assessment of the potential effects of neonicotinoids on frogs.

Although pesticides in general have been proposed as a contributing factor in amphibian population declines (Davidson 2004; Sparling et al. 2015; Grant et al. 2016), we found no evidence to suggest that clothianidin or thiamethoxam would directly reduce survival or fitness‐related traits in 2 amphibian species. This suggests that, under the specific experimental conditions in the present study, clothianidin and thiamethoxam had no effect on the conventional life‐history endpoints selected. Furthermore, based on the present study and some previous work with wood frogs using a similar mesocosm design (Robinson et al. 2017), it appears that wood frogs are more sensitive to handling stress or the mesocosm conditions during the early tadpole stages than leopard frogs. Survival in our wood frog control mesocosms was 67%, and in the similar wood frog mesocosm study survival was 65% where these mesocosms were established 20 d prior to adding tadpoles (Robinson et al. 2017) compared to 7 d in the present study. For leopard frogs, where the mesocosms were established only 2 d prior to adding tadpoles, survival was 85% in the controls. Although the percent survival for wood frogs in control mesocosms was less than ideal, it was within the range reported from other amphibian mesocosm studies, where percent survival of controls can range from 15 to 99% (Egea‐Serrano et al. 2012). Hence, wood frogs may be more sensitive than leopard frogs when using mesocosms, but their survival is within the upper range found in other mesocosm studies using amphibians.

Amphibians are widely considered to be bioindicators of ecosystem health in aquatic environments associated with agriculture (Hopkins 2007); hence, assessing the effects of contaminants on conventional life‐history traits is important for establishing a baseline of the potential toxicity of pesticides. Although there have been only a few studies assessing the toxicity of neonicotinoids to amphibians, minimal detrimental effects have been reported at environmentally relevant concentrations. Rios et al. (2017) found that bullfrog tadpoles (*Rana catesbeiana*) exposed to 0.1 µg/L of imidacloprid for 8 wk had no effect on antibody response or immune response. Hrynyk et al. (2018) found no mortality of *Xenopus* tadpoles exposed to 1 or 500 µg/L of imidacloprid after 40 d of exposure and that exposure to imidacloprid also reduced tadpole mortality following exposure to the wild‐type frog virus 3 (*Ranavirus*). Chronic exposure (21 d) to 0.25 to 1 mg/L of clothianidin elicited no sublethal effects on swimming activity of northern leopard frog tadpoles, and no mortality was observed at the saturation point of formulated clothianidin within 48 h (48‐h median lethal concentration [LC50], Arena, ~327 mg/L; Miles et al. 2017). In a recent study related to the present study, reduced mortality and a slight delay in metamorphosis in wood frogs were reported with exposure to imidacloprid at 10 and 100 µg/L but not to thiamethoxam (1, 10, 100 µg/L; Robinson et al. 2017). The authors suggested that the slight delay in metamorphosis was likely not ecologically relevant (Robinson et al. 2017). These studies, combined with the absence of effects with clothianidin and thiamethoxam at concentrations of 2.5 and 250 µg/L observed in the present study, support the premise that amphibians appear to be relatively insensitive to direct neonicotinoid exposure and, hence, are less sensitive to neonicotinoids than the targeted invertebrates (Simon‐Delso et al. 2015).

Although amphibians appear to be insensitive to the direct effects of neonicotinoids in the absence of other stressors, added stressors, particularly at longer exposure times than typical LC50 tests, may result in marked increases in pesticide toxicity (e.g., Relyea and Mills 2001). Furthermore, combining the effects of neonicotinoids along with concurrent environmental contaminants or stressors may be important in establishing the toxicity of neonicotinoids in more realistic scenarios. For example, Pochini and Hoverman (2017) found that prior *Ranavirus* infection increased pesticide toxicity in wood frogs, specifically reducing the lethal concentration of thiamethoxam by 55% (from ~2.2 to ~1 mg/L). Finally, neonicotinoids could have indirect effects on amphibians by reducing prey abundance because insects have been found to be directly affected at much lower concentrations and well within the concentration range tested in the present study (Bartlett et al. 2018; Raby et al. 2018); this is similar to concerns for aerial insectivores, such as tree swallows (Gibbons et al. 2014). Although the effects of individual neonicotinoids in controlled laboratory environments have provided important insights on their aquatic toxicology, interactive effects between chemical contamination and additional stressors such as predation or parasitism could provide a more comprehensive understanding of the risks of neonicotinoids in whole ecosystems (Marcogliese and Pietrock 2011).

Our nominal concentrations of 2.5 and 250 µg/L were well below the reported LC50s of a similar neonicotinoid (imidacloprid, range 52.6–366 mg/L) for other amphibians (Feng et al. 2004; Sánchez‐Bayo 2012; Gibbons et al. 2014; Pérez‐Iglesias et al. 2014; Ruiz De Arcaute et al. 2014), and our lower dose was within the upper range of detected concentrations in ecosystems where wood frogs and leopard frogs, or related species, breed and reproduce (Morrissey et al. 2015). Neonicotinoids are soluble in water and degrade when exposed to ultraviolet light via sunlight (Bonmatin et al. 2015). In our mesocosms, we found that concentrations of clothianidin and thiamethoxam increased over time, suggesting that the compounds were not degrading as rapidly as predicted (Supplemental Data, Table S3). The reported half‐lives in water are <1 and 0.2 to 1.5 d for clothianidin and thiamethoxam, respectively (outdoor mesocosms; see Lu et al. 2015 for details); however, measurable concentrations are often observed persisting in the environment for up to 1 yr postapplication (Lu et al. 2015; Morrissey et al. 2015). A mesocosm experiment demonstrated that thiamethoxam degradation was negligible at water depths of >8 cm (Lu et al. 2015). Even though we reduced the dosing frequency of mesocosms by half compared to Robinson et al. (2017), we still found that neonicotinoids persisted longer than expected, which can likely be attributed to the reduced light penetration from the 40% shade cloth over the mesocosms (similar to canopy shading in the environment), the depth of the water column, the increased tannins as the leaf litter decomposed, and the increase in phytoplankton as the mesocosm community developed. Given that the aqueous persistence of neonicotinoids is highly dependent on numerous abiotic factors such as lighting and water quality, additional research into the persistence of neonicotinoids in the aquatic environment typical of breeding amphibians would better indicate more realistic exposure scenarios.

We predicted that at our test concentrations there would be no effect of neonicotinoid exposure on amphibian survival; however, under chronic exposure, underlying sublethal effects may become apparent. Exposure to amphibians during development from a tadpole to a frog in mesocosms was chosen as the route of exposure because neonicotinoids are highly water‐soluble and amphibians depend on surface waters associated with agriculture for breeding and development. Although there were no individual direct effects of thiamethoxam and clothianidin seen in the present study, we suggest that additional research is warranted on the interaction of neonicotinoids with other environmental stressors, including exposure to pesticide (or other contaminant) mixtures, or natural stressors such as predators or parasites. Examining the interactions of neonicotinoids in a broader context of other ecosystem biotic and abiotic drivers of amphibian populations and expanding assessments to include indirect effects (e.g., effects on amphibian predators or prey) would help to fully understand their effects on amphibians and to determine their relevance to amphibian development and population dynamics.

## Supplemental Data

The Supplemental Data are available on the Wiley Online Library at DOI:10.1002/etc.4511.

## Data Accessibility

Raw data have been included as Supplemental Data.

## Supporting information

This article includes online‐only Supplemental Data.

Supporting information.
