# Supplementary material for: Assessment of Sublethal Effects of Neonicotinoid Insecticides on the Life‐History Traits of 2 Frog Species
Source: Environ Toxicol Chem. 2019 Aug 6;38(9):1967–77. doi: 10.1002/etc.4511 (PMC7322800; doi:10.1002/etc.4511)
Supplement: Supplementary file 1 — Supporting information. [file ETC-38-1967-s001.docx]

**Supplementary Information**

**Assessment of sub-lethal effects of neonicotinoid insecticides on the life-history traits of two frog species**

Robinson, S.A., Richardson, S.D., Dalton, R.L., Maisonneuve, F., Bartlett, A.J., de Solla, S.R., Trudeau, V., Waltho, N.

**Results**

**Neonicotinoid Analyses**

Although all quality assurance and control measures were accurate and calibration curves were confirmed, we found the percent recoveries compared to expected nominal concentrations for a subset of our samples were low, particularly compared to a previous study that followed a similar experimental design and used the same laboratory for chemical analyses; Robinson et al. 2017). The mean percent recovery in the current study was 40% (± 18%, SD) for samples collected between May 14^th^ and July 2^nd^ (Table S2; samples were analyzed by the laboratory July 3^rd^ to 10^th^). However, percent recovery increased for samples that were collected between July 8^th^ and 28^th^ (Table S2) with a mean recovery of 129% (± 12%, SD; samples were analyzed by the laboratory July 13^th^ to 30^th^). After a thorough investigation, a faulty batch of Acrodisc syringe filters (0.45 µm PVDF membrane, Product number: WAT2008274, from Waters Corporation, Milford, MA, USA) was identified as the source of the low recoveries. The problem with the filters was not detected initially because the samples were quantified with the external standard quantification method (i.e., an internal standard was not used). During our investigation, we compared 5 different sources of filters, including those from different batches and different companies, and we found that only the one particular batch of filters retained over 50% of the neonicotinoids. Unfortunately, we did not have any samples remaining from the mesocosm exposures to be re-analyzed; however, we had two stock solutions available (from our second set) and samples from two separate laboratory studies (Bartlett et al., 2019 and Prosser et al. 2016) that had been chemically analyzed at the same time by the same laboratory (Laboratory Services, NWRC). These stock solution samples and samples from the laboratory studies were analyzed by Laboratory Services at NWRC and a second analytical laboratory located at the Ontario Ministry of the Environment, Conservation and Parks (OMOECP). High percent recoveries were obtained from the analysis of the second set stock solutions from both Laboratory Services and OMOECP analyses (97-128%, respectively; Table S3). The samples from the laboratory studies also had high percent recoveries from both Laboratory Services and OMOECP analyses (90-105%, respectively; Table S3). Furthermore, filtered samples from the original analysis (where faulty filters were used) of the first set stock solutions and the laboratory study by Bartlett et al. (2019) were available for re-testing to confirm the reduced recovery was most likely the filters and not an issue with the stock solution preparation, calibration curve, other equipment or protocol. These filtrates were re-injected for analysis and they still had low percent recovery (34-43%; Table S3). Therefore, we suggest our measured concentrations (i.e., exposure concentrations) are closer to the expected nominal concentrations than what was measured and reported in Table S2 from May 14^th^ to July 2^nd^ and that the filter issue was resolved and measured concentrations are more reliable from July 8^th^ to 28^th^ in Table S2. Three previous studies conducted by corresponding research teams (Robinson et al. 2017, Prosser et al. 2016, Bartlett et al. 2019) and chemical analysis by the same laboratory (i.e., Laboratory Services, NWRC) have reported measured concentrations close to the expected nominal concentrations; hence, this faulty filter issue was an unfortunate and unpredictable occurrence.

**Table S1.** Weekly mean mesocosm water quality parameters ± standard deviation (temperature (°C), dissolved oxygen (mg/L) and pH) by treatment (CLO = clothianidin, THI = thiamethoxam) and sampling date during the experimental period from May 13 to July 30, 2015 for wood frogs (*Lithobates sylvaticus*) and from May 21 to July 22, 2015 for leopard frogs (*Lithobates pipiens*). Data are reported as mean ± standard deviation. Nominal concentrations of 2.5 µg/L and 250 µg/L are denoted in the column headings for each treatment (e.g. CLO-2.5 = clothianidin 2.5 µg/L).

**Date Control CLO-2.5 CLO-250 THI-2.5 THI-250**

**Temperature (mean °C ± SD)**

*Wood frogs (L. sylvaticus)*

May 13^b^ 16.7 ± 0.2 16.4 ± 0.5 16.4 ± 0.5 16.5 ± 0.4 16.5 ± 0.3

June 18 23.1 ± 0.7 23.1 ± 0.5 22.9 ± 0.6 23.5 ± 0.6 22.1 ± 0.4

June 24^b^ 26.0 ± 0.7 24.4 ± 0.2^a^ 25.3 ± 0.3 25.3 ± 0.5 25.4 ± 0.2

June 30^b^ 22.0 ± 0.4 21.5 ± 0.2 21.6 ± 0.2 21.6 ± 0.4 23.0 ± 2.8

*Leopard frogs (L. pipiens)*

May 21^c^ 12.7 ± 0.3 12.6 ± 0.5 12.5 ± 0.3 12.9 ± 0.1 12.7 ± 0.1

June 18 24.2 ± 0.3 24.2 ± 0.4 24.2 ± 0.5 24.0 ± 0.4 23.9 ± 0.5

June 24^c^ 24.4 ± 0.5 23.4 ± 0.6 24.4 ± 0.6 24.2 ± 0.6 24.7 ± 0.6

June 30 21.1 ± 0.3 20.5 ± 0.5 21.1 ± 0.5 21.0 ± 0.4 21.2 ± 0.6

July 08 23.2 ± 0.2 22.5 ± 0.4 23.2 ± 0.4 23.2 ± 0.4 23.8 ± 0.2

July 15 21.0 ± 0.3 20.2 ± 0.5 20.9 ± 0.4 20.7 ± 0.2 21.2 ± 0.7

July 22^c^ 24.0 ± 0.3 23.4 ± 0.5 23.9 ± 0.4 24.0 ± 0.2 24.0 ± 0.4

**Dissolved oxygen (mean mg/L ± SD)**

*Wood frogs (L. sylvaticus)*

May 13^b^ 9.1 ± 1.1 9.8 ± 0.9 9.6 ± 1.3 9.8 ± 0.7 9.2 ± 1.0

June 18 8.9 ± 0.3 8.9 ± 0.3 8.7 ± 0.2 8.8 ± 0.3 8.9 ± 0.2

June 24^b^ 9.0 ± 0.2 8.9 ± 0.2 8.9 ± 0.4 9.0 ± 0.2 8.7 ± 0.2

June 30^b^ 9.5 ± 0.2 9.3 ± 0.2 9.5 ± 0.2 9.5 ± 0.1 9.4 ± 0.0

*Leopard frogs (L. pipiens)*

May 21^c^ 8.7 ± 0.4 9.2 ± 0.7 9.3 ± 0.3 8.5 ± 0.7 9.2 ± 0.6

June 18 7.8 ± 0.5 8.4 ± 0.2 8.0 ± 0.4 7.7 ± 0.5 7.4 ± 1.3

June 24^c^ 8.4 ± 0.4 8.8 ± 0.2 8.5 ± 0.1 8.4 ± 0.2 8.3 ± 0.3

June 30 9.0 ± 0.2 9.4 ± 0.1 9.2 ± 0.2 9.1 ± 0.2 9.3 ± 0.1

July 08 8.8 ± 0.6 9.1 ± 0.2 8.9 ± 0.3 8.9 ± 0.2 9.1 ± 0.2

July 15 6.7 ± 1.1 7.4 ± 0.4 6.9 ± 0.7 6.6 ± 0.5 7.2 ± 0.7

July 22^c^ 6.2 ± 0.7 6.7 ± 0.2 6.1 ± 0.4 6.2 ± 1.0 6.2 ± 0.5

**pH (mean ± SD)**

*Wood frogs (L. sylvaticus)*

May 13^b^ 7.8 ± 0.1 7.9 ± 0.2 7.9 ± 0.1 8.0 ± 0.1 7.9 ± 0.1

June 18 7.5 ± 0.2 7.7 ± 0.1 7.8 ± 0.1 7.7 ± 0.1 7.9 ± 0.2

June 24^b^ 7.7 ± 0.2 8.3 ± 0.1 ^a^ 8.0 ± 0.1 8.1 ± 0.1^a^ 7.6 ± 0.1

June 30^b^ 7.1 ± 0.1 7.9 ± 0.4 ^a^ 7.1 ± 0.1 7.2 ± 0.1 7.0 ± 0.1

*Leopard frogs (L pipiens)*

May 21^c^ 8.2 ± 0.1 8.2 ± 0.2 8.1 ± 0.2 8.0 ± 0.1 8.1 ± 0.2

June 18 7.7 ± 0.0 7.8 ± 0.0 7.7 ± 0.1 7.8 ± 0.0 7.8 ± 0.0

June 24^c^ 8.0 ± 0.1 8.2 ± 0.0 ^a^ 8.0 ± 0.1 8.1 ± 0.1 7.9 ± 0.2

June 30 7.4 ± 0.3 8.2 ± 0.0 7.2 ± 0.1 7.6 ± 0.4 7.0 ± 0.0

July 08 8.1 ± 0.2 7.9 ± 0.2 7.6 ± 0.3 8.1 ± 0.1 8.4 ± 0.1

July 15 7.8 ± 0.2 7.8 ± 0.1 7.8 ± 0.2 7.8 ± 0.1 8.0 ± 0.1

July 22^c^ 7.8 ± 0.1 7.8 ± 0.2 7.8 ± 0.1 7.7 ± 0.1 8.0 ± 0.1

^a^ Significantly different from controls at *p* < 0.05 Kruskall-Wallis.

^b^ Early (May 13^th^), mid (June 24^th^) and late (June 30^th^) time periods selected to examine differences in abiotic conditions for wood frogs.

^c^ Early (May 21^st^), mid (June 24^th^) and late (July 22^nd^) time periods selected to examine differences in abiotic conditions for leopard frogs.

**Table S2.** Concentrations (µg/L) of clothianidin (CLO) and thiamethoxam (THI) by treatment and sampling date during the experimental period from May 14 to July 2, 2015 for wood frogs (*Lithobates sylvaticus*) and from May 28 to July 28, 2015 for leopard frogs (*Lithobates pipiens*). Initial targeted nominal concentrations were 1 µg/L and 100 µg/L (corresponds to May 14^th^ concentrations only), but thereafter nominal concentrations were changed to 2.5 µg/L and 250 µg/L and are denoted in the column headings for each treatment (e.g. CLO-2.5 = clothianidin 2.5 µg/L). Because of faulty filters used during the chemical analysis, the measured concentrations were less than actual exposure concentrations until July2^nd^ when we think the filter issue was resolved, and thus measured concentrations were more reliable.

______________________________________________________________________________________________________

**Treatment concentration (µg/L; mean ± SD)**

**Date Control CLO-2.5 Nominal (%) CLO-250 Nominal (%) THI-2.5 Nominal (%) THI-250 Nominal (%)**

______________________________________________________________________________________________________

***Wood frogs (L. sylvaticus)***

May 14^ab^ <LOD 0.4±0.02 41.0 22.0±1.1 22.0 0.4±0.01 44.0 32.1±3.1 32.1

May 27^cd^ <LOD 1.0 38.4 56.0 22.4 0.8 30.4 86.4 34.6

June 3^ce^ <LOD 0.8 33.6 52.0 20.8 0.7 26.0 89.3 35.7

June 10^cd^ <LOD 1.6 64.0 125.0 50.0 1.7 66.4 145.5 (0.6)^f^ 58.2

June 17^ce^ <LOD 0.8 31.2 117.0 46.8 1.45 58.0 129.5 (0.79)^f^ 51.8

June 24^cd^ <LOD 0.6 22.8 223.0 89.2 1.63 65.2 225.0 (1.0)^f^  90.0

July 2^g^ <LOD 1.2±0.6 46.8 190.3±12.5 76.1 - - 157.1±24.3 62.8

July 2^h^ <LOD - - 249.4±11.5 99.8 2.3±0.2 93.2 - -

**Leopard frogs (*L. pipiens*)**

May 28^b^ 0.04 (THI) 0.7±0.1 26.4 44.9±2.2 17.9 0.7±0.1 28.4 73.2±3.2 29.3

June 3^cd^ <LOD 0.6 23.6 40 .0 16.0 0.7 27.2 78.9 31.5

June 10^cd^ <LOD 1.6 64 .0 129.0 51.6 1.6 63.6 142.5 57.0

June 17^ce^ <LOD 1.0 40.0 104.0 41.6 1.4 55.2 114.0 45.6

June 24^cd^ <LOD 1.7 68.0 215.0 86.0 1.8 72.8 173.5 69.4

July 2^ce^ <LOD 1.4 56.0 - - - - 163.0 65.2

July 2^ch^ - - - 265.0 106.0 2.4 96.8 - -

July 8^cd^ <LOD 2.8 112.0 294.0 117.6 3.13 125.2 287.5 (1.3)^f^ 115.0

July 15^ce^ <LOD 3.2 128.0 347.0 138.8 3.2 126.0 335.0 (1.4)^f^ 134.0

July 22^ci^ <LOD 3.6 144.0 339.0 135.6 3.7 146.0 298.0 (2.1)^f^ 119.2

July 28^gj^ <LOD 3.5±0.3 138.8 325.7±14.5 130.3 3.4±0.14 137.2 285.5±20.5 114.2

(1.9±0.2)^f^

______________________________________________________________________________________________________

^a^ On May 14^th^, the nominal concentration was 1 µg/L and 100 µg/L for clothianidin and thiamethoxam, as this was the initial targeted nominal concentration prior to any adjustments based on chemical analyses.

^b^ All 5 mesocosms per treatment were sampled one hour after the first dose was applied on May 14^th^ and 28^th^ for wood frogs and leopard frogs, respectively, to establish initial exposure concentrations

^c^ One random replicate mesocosm per treatment was analyzed

^d^ Concentrations represent samples collected one week after dosing

^e^ Concentrations represent samples collected two weeks after dosing

^f^ Clothianidin is a breakdown product of thiamethoxam and the concentrations are provided in parentheses after the associated THI-250 concentrations

^g^ All 5 mesocosms per treatment were sampled at the end of the experimental period (July 2^nd^ for wood frogs and July 28^th^ for leopard frogs) to establish final exposure concentrations

^h^ Samples analyzed by laboratory July 16^th^ after faulty filter issue resolved (CLO-250: n = 2; THI-2.5: n = 5)

^i^ Concentrations represent samples collected three weeks after dosing

^j^ Concentrations represent samples collected four weeks after dosing

SD = Standard deviation

**Table S3.** Concentrations (µg/L) and percent nominal (%Nom) concentrations of clothianidin (CLO) and thiamethoxam (THI) from first and second sets of stock solutions used for mesocosm exposures. The second set of stock solutions was analyzed by two independent laboratories (Laboratory Services at the National Wildlife Research Centre (NWRC-lab) and a laboratory at the Ontario Ministry of the Environment, Conservation and Parks (OMOECP-lab)). Chemical analyses with percent nominal concentrations for clothianidin and thiamethoxam from two independent studies (Bartlett et al. 2019 and Prosser et al. 2016) are also presented to further support the hypothesis that a faulty lot of filters used during chemical analyses was responsible for the reduced measured concentrations. Based on these re-analyses as well as the high recoveries, we suggest nominal concentrations were reflective of the exposure concentrations.

_____________________________________________________________________________________________________

**Clothianidin Thiamethoxam**

**NWRC-lab OMOECP-lab NWRC-lab OMOECP-lab**

**Nominal [CLO] %Nom [CLO] %Nom [THI] %Nom [THI] %Nom**

**_____________________________________________________________________________________________________**

**Stock solutions**

First set 30 000 13 500 45 - - 10 100 34 - -

3 000 000 1 050 000 35 - - 1 014 000 34 - -

Re-analysis of originals^a^ 30 000 12 800 43 - - 13 000 42 - -

3 000 000 1 012 000 34 - - 1 273 000 42 - -

Second set^b^ 30 000 38 500 128 34 000 113 - - - -

3 000 000 - - - - 3 390 000 113 2 900 000 97

**Bartlett et al. 2019**

Original analyses 0.156 0.07 42 - - - - - -

Re-analysis of originals^a^ 0.156 0.06 40 - - - - - -

Additional samples^c^ 0.31 0.30 97 0.29 94 - - - -

0.31 0.33 106 0.30 97 - - - -

5 4.95 99 4.60 92 - - - -

**Prosser et al. 2016**

Original analyses 3.3 1.35 41 - - 1.40 42 - -

33.0 12.1 37 - - 13.6 31 - -

Additional samples^c^ 5.0 5.23 105 4.5 90 - - - -

5.1 5.00 98 4.9 96 - - - -

51 48.9 96 47.0 92 - - - -

8.9 - - - - 8.86 100 8.00 90

89 - - - - 89.0 100 78.0 88

**______________________________________________________________________________________________________** ^a^ Consisted of re-injecting the filtrate from the original vials that were prepared by diluting the sample with reverse osmosis water and filtering through the Acrodisc syringe filter (0.45 µm PVDF membrane) that we think was from a faulty batch of filters used for a subset of the current studies chemical analyses and some of the samples from Prosser et al. 2016 and Bartlett et al. 2019. The original samples themselves were not reanalyzed.

^b^ Samples are from the same experiment and are directly comparable to the other samples, as they were prepared and stored the same as the first set stock solution; however, these chemical analyses were conducted with a new batch of filters (i.e., Millex syringe filters (0.45 µm PVDF membrane).

^c^ Samples are from the same experiment and are directly comparable to the other samples, as they were prepared, collected and stored on the same day using the same stock solution; however, these chemical analyses were conducted with a new batch of filters (i.e., Millex syringe filters (0.45 µm PVDF membrane).

**Table S4.** The survival (mean percentage ± standard deviation) and proportion (Prop. %; mean percentage ± standard deviation) of female to male (gonadal gross morphology) wood frogs (*Lithobates sylvaticus*) and northern leopard frogs (*Lithobates pipiens*) after chronic exposure to clothianidin (CLO-2.5, CLO-250) or thiamethoxam (THI-2.5, THI-250) in outdoor mesocosms. Sample size for sexual differentiation for each treatment is indicated (N).

Treatment ^a^ Wood frogs (*L. sylvaticus*) Leopard frog (*L. pipiens*)

Survival^b^ Female Male Prop. F^b^ N Survival^b^ Female Male Prop. F^b^ N

Control 67 ± 8 21 46 32 ± 4 67 85 ± 7 34 31 56 ± 17 65

CLO-2.5 75 ± 5 31 43 42 ± 12 74 83 ± 13 35 19 70 ± 18 54

CLO-250 71 ± 15 20 49 30 ± 10 69 89 ± 8 41 21 64 ± 19 62

THI-2.5 74 ± 17 30 43 43 ± 22 73 83 ± 7 42 22 68 ± 19 64

THI-250 70 ± 6 25 44 37 ± 9 69 91 ± 5 45 28 61 ± 5 73

^a^ Nominal treatment concentrations were 2.5 µg/L and 250 µg/L for clothianidin (CLO-2.5 and CLO-250) and thiamethoxam (THI-2.5 and THI-250).

^b^ Mean percentage across the 5 replicate blocks per treatment ± standard deviation (% ± SD)

**Table S5.** Developmental morphology (i.e., snout-to-vent length, body mass) of wood frogs (*Lithobates sylvaticus*) and northern leopard frogs (*Lithobates pipiens*) after chronic exposure to clothianidin (CLO-2.5, CLO-250) or thiamethoxam (THI-2.5, THI-250) in outdoor mesocosms. Sample size for each treatment is indicated (N).

Body Measurement^a^ Wood frogs (*L. sylvaticus*) Leopard frog (*L. pipiens*)

Median (25-75% interquartile range) N Median (25-75% interquartile range) N

Snout-to-vent length (mm)

Control 22.2 (21.6-23.2) 66 26.9 (24.7-28.9) 64

CLO-2.5 21.3 (20.7-22.3) 75 26.7 (24.3-28.4) 51

CLO-250 22.0 (21.3-22.7) 68 26.3 (25.1-27.8) 61

THI-2.5 22.0 (21.5-22.6) 74 26.3 (25.0-28.9) 65

THI-250 22.5 (21.6-23.2) 70 27.1 (25.3-29.1) 74

Body mass (g)

Control 0.8 (0.7-0.9) 67 1.2 (0.9-1.4) 65

CLO-2.5 0.7 (0.6-0.8) 75 1.1 (0.8-1.3) 52

CLO-250 0.8 (0.7-0.9) 70 1.1 (0.9-1.3) 62

THI-2.5 0.8 (0.7-0.9) 74 1.1 (0.9-1.5) 65

THI-250 0.8 (0.7-0.9) 70 1.2 (1.0-1.4) 74

Days to Metamorphosis

Control 46 (45-49) 67 55 (54-60) 65

CLO-2.5 46 (45-49) 75 55 (53-62) 54

CLO-250 46 (46-49) 71 60 (55-64) 62

THI-2.5 46 (45-47) 74 55 (53-60) 65

THI-250 49 (46-49) 70 55 (54-60) 59

Stage of Development

Control ^b^  40 (37-42) 20

CLO-2.5 ^b^ 40 (40-42) 29

CLO-250 ^b^ 40 (40-42) 26

THI-2.5 ^b^ 40 (39-42) 18

THI-250 ^b^ 41 (40-44) 17

^a^ Nominal treatment concentrations were 2.5 µg/L and 250 µg/L for clothianidin (CLO-2.5 and CLO-250) and thiamethoxam (THI-2.5 and THI-250).

^b^ Life history trait not evaluated.

**
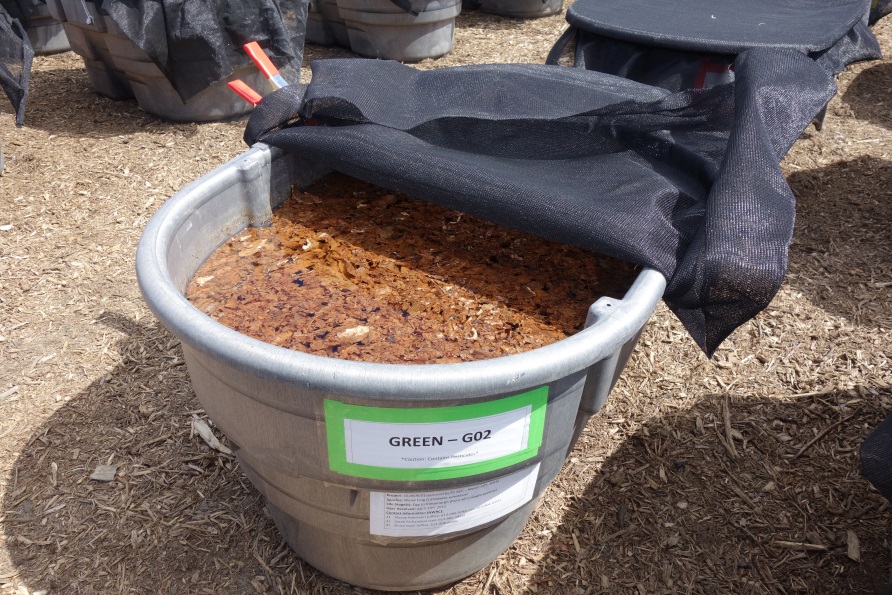
**

**Figure S1.** Rubbermaid® mesocosms filled with 300 L tap water and 333.3 ± 5.0 g of mixed deciduous leaf litter (settled at bottom within 1 day), covered with 40% shadecloth to prevent unwanted organisms from entering or frogs from escaping the mesocosms.

N

**
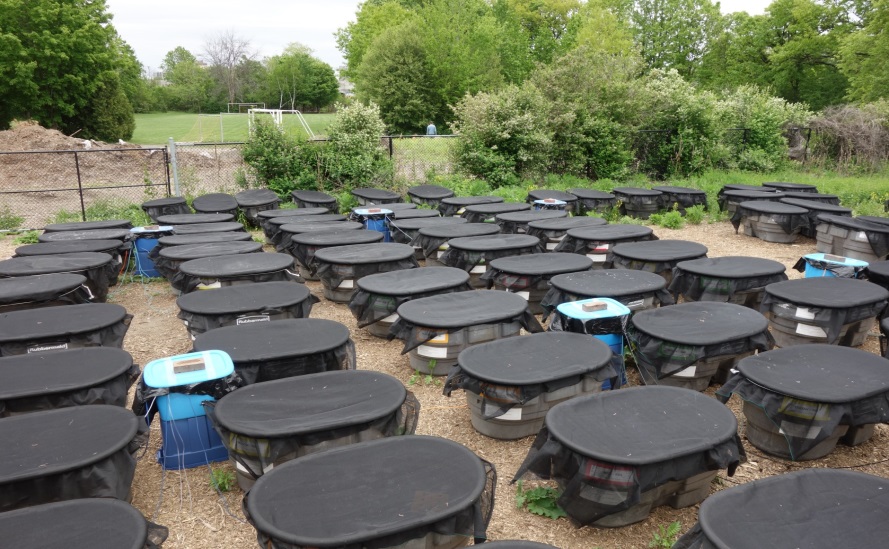
**

**Figure S2.** Random block design of experimental mesocosms running North to South to account for possible shading effects of neighboring mesocosms.


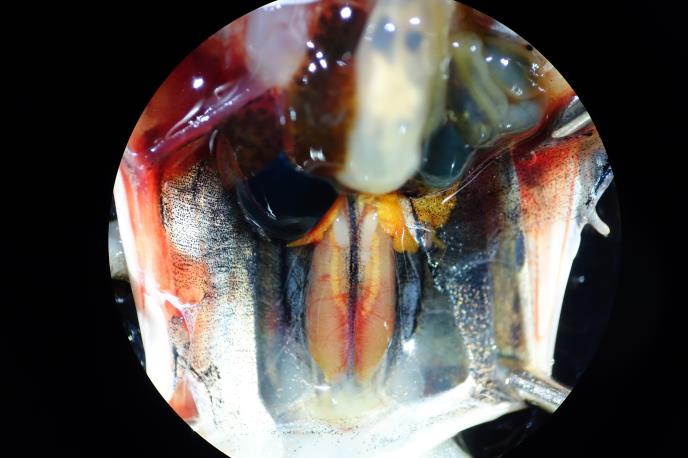

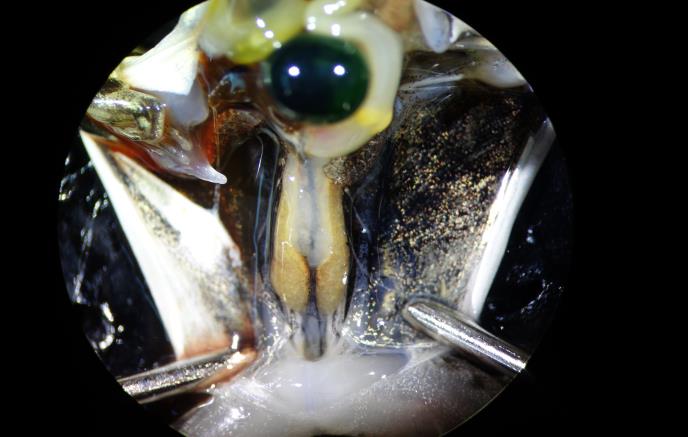


**Figure S3.** Gonadal gross morphology of emerging wood frog *(Lithobates sylvaticus)* metamorphs (male on left vs. female on right). Photograph taken through lens of dissecting microscope at 0.65x magnification.

**Reference list**

Bartlett AJ, Hedges AM, Intini KD, Brown LR, Maisonneuve FJ, Robinson SA, Gillis PL, de Solla SR. 2019. Acute and chronic toxicity of neonicotinoid and butenolide insecticides to the freshwater amphipod, *Hyalella azteca. Ecotoxicol Environ Saf* 175:215-223; DOI: 10.1016/j.ecoenv.2019.03.038.

Prosser RS, de Sola SR, Holman EAM, Osborne R, Robinson SA, Bartlett AJ, Maisonneuve FJ, Gillis PL. 2016. Sensitivity of the early-life stages of freshwater mollusks to neonicotinoid and butenolide insecticides. *Environ Pollut* 218:428-435; DOI: 10.1016/j.envpol.2016.07.022.

Robinson SA, Richardson SD, Dalton RL, Maisonneuve F, Trudeau VL, Pauli BD, Lee-Jenkins SS. 2017. Sublethal effects on wood frogs chronically exposed to environmentally relevant concentrations of two neonicotinoid insecticides. *Environ Toxicol Chem* 36:1101–1109; DOI:10.1002/etc.3739.
